# Efficiency of Dendritic Cell Vaccination against B16 Melanoma Depends on the Immunization Route

**DOI:** 10.1371/journal.pone.0105266

**Published:** 2014-08-14

**Authors:** Fanny Edele, Jan C. Dudda, Eva Bachtanian, Thilo Jakob, Hanspeter Pircher, Stefan F. Martin

**Affiliations:** 1 Allergy Research Group, Department of Dermatology, Medical Center - University of Freiburg, Freiburg, Germany; 2 Institute for Medical Microbiology and Hygiene, Medical Center - University of Freiburg, Freiburg, Germany; Baylor College of Medicine, United States of America

## Abstract

Dendritic cells (DC) presenting tumor antigens are crucial to induce potent T cell-mediated anti-tumor immune responses. Therefore DC-based cancer vaccines have been established for therapy, however clinical outcomes are often poor and need improvement. Using a mouse model of B16 melanoma, we found that the route of preventive DC vaccination critically determined tumor control. While repeated DC vaccination did not show an impact of the route of DC application on the prevention of tumor growth, a single DC vaccination revealed that both the imprinting of skin homing receptors and an enhanced proliferation state of effector T cells was seen only upon intracutaneous but not intravenous or intraperitoneal immunization. Tumor growth was prevented only by intracutaneous DC vaccination. Our results indicate that under suboptimal conditions the route of DC vaccination crucially determines the efficiency of tumor defense. DC-based strategies for immunotherapy of cancer should take into account the immunization route in order to optimize tissue targeting of tumor antigen specific T cells.

## Introduction

Dendritic cells play a central role in the initiation of immune responses. Since tumor antigen-bearing DC are capable to induce protective T cell mediated immune responses several strategies have been developed using DC vaccines for cancer therapy [1]–[3]. The most promising strategy might be the *ex vivo* generation of DC from blood or bone marrow-derived precursors, as these can be fully characterized and an array of parameters can be manipulated to optimize the DC vaccine. These include DC subset differentiation, activation status as well as antigen delivery and presentation. It has been described that injection of mature DC pulsed with tumor associated antigens (TAA) or tumor lysate were more potent in generating antigen-specific T cell responses compared to immature DC [4],[5]. DC vaccination was used in patients with different types of cancer [6]–[11] and in phase 1 trials it was shown that *ex vivo* generated peptide-pulsed DC induced antigen-specific immune responses [4], [12]. Unfortunately immune responses were transient and clinical outcomes have been poor. The best results were obtained in patients with melanoma at cutaneous or lymphatic sites [13]. Recently the FDA has approved the first DC-based vaccine against human metastatic prostate cancer [14].

It was shown that different injection routes of DC result in activation of T cells in different lymphoid organs. For example i.v. injected DC efficiently enter the spleen [15] whereas subcutaneously (s.c.) injected DC access peripheral LN draining the injection area [16]–[19]. It also was described that the magnitude of the primary immune response directly correlates with the infiltration of DC and antigen-specific naive T cells into individual LN [20]. This was demonstrated in a mouse model of tumor control by injection of varying numbers of bone marrow-derived DC. The number of injected DC correlated with the number of DC infiltrating the draining LN, with the number of antigen specific CD8^+^ T cells in the same LN and the decrease in tumor size. Furthermore it was described that T cells activated by DC isolated from different tissues express different homing receptors necessary for entering peripheral organs. T cells activated with DC isolated from mesenteric LN (mLN) express the small intestine homing receptors chemokine receptor 9 (CCR9) and α4β7 integrin. In contrast T cells activated with Langerhans cells isolated from the epidermis express the skin homing receptors E-selectin ligand (E-lig) [21]. We and others have previously shown that the peripheral tissue microenvironment has an impact on the capacity of the DC to induce homing receptors on T cells [22]–[24]. Therefore the microenvironment of the DC origin licenses DC to induce homing receptors on T cells in the draining lymph nodes. This was further shown by Calzascia and colleagues by transplanting tumors expressing two different tumor antigens into different sites, intracerebral and subcutaneous, in the neck [25]. Both sites are drained into the cervical LN where T cell homing receptors were analyzed. In this cervical LN, DC were found to have immigrated from the two different tumor sites presenting the respective tumor antigens. These activated DC induced the homing receptor profiles on T cells corresponding to their tissue of origin, the brain and the skin, in the same lymph node. Therefore multiple imprinting programs occurred in the same LN. The authors concluded that the identity of a given LN is not essential in determining homing receptor imprinting but rather the site of antigen uptake by DC [25]. In contrast to this another study showed that the microenvironment of a given LN also has an impact on the induction of homing receptor imprinting on T cells [23]. This was done by transplanting peripheral LN (pLN) into the gut mesenteries. These transplanted LN fail to support the generation of gut-homing T cells, even though gut-derived DC enter the transplants and prime T cells [23].

All these reports led us to the hypothesis that the correct immunization route might be critical for the outcome of tumor defense in DC-based immunotherapy by determining the homing behavior of tumor antigen specific T cells. Here we show that the route of DC injection is crucial for the outcome of protective DC vaccination in a melanoma model in the mouse. We have established a preventive protocol for DC vaccination against melanoma growth. This revealed that only the i.c. injection of bone marrow-derived DC (BM-DC) which were loaded with the LCMV-derived peptide GP33, primed T cells to up-regulate the homing receptor E-selectin ligand necessary for the migration into the skin. In comparison after i.p. injection of peptide-loaded DC, effector T cells up-regulate homing receptors related to migration to the small intestine (α4β7 and CCR9). Additionally in contrast to the i.p. administration route the i.c. vaccination led to an enhanced proliferation and activation state of cytotoxic T cells. In consequence only i.c. injection of BM-DC resulted in long-term prevention of tumor growth. These data show that the immunization route used for DC vaccination should be considered in order to optimize the efficiency of the T cell response to the tumor in immunotherapy.

## Materials and Methods

### Mice

C57BL/6 and TCR-transgenic Thy1.1 congenic P14 mice (P14.1.1) [26], [27] expressing a TCR specifically recognizing the lymphocytic choriomeningitis virus (LCMV)-derived peptide GP33 [28] were provided by the breeding facility of the Medical Center - University of Freiburg, Germany. Also C57BL/6 mice were purchased from Charles River Laboratories. All of the experimental procedures were in accordance with institutional, state and federal guidelines on animal welfare. All protocols for animal experiments were approved by the Regierungspräsidium Freiburg and supervised by the Animal Protection Representatives of the Medical Center - University of Freiburg.

### Cell lines and peptides

Two melanoma cell lines were used. The parental cell line: B16.F10 (ATCC number: CRL-6475) and a GP33 expressing cell line: B16.F10_GP33_ [29]. 1×10^6^ cells were injected subcutaneously (s.c.) into the left and the right flank of C57BL/6 mice, respectively.

The HPLC purified LCMV peptide GP33 (KAVYNFATM), binding to H-2D^b^ (Hermann GbR, Freiburg, Germany) was used in a final concentration of 1 µM to pulse DC. As control peptide an adenovirus peptide (Ad5E1A SGPSNTPPEI) was used in a final concentration of 0.1 µM.

### Isolation and preparation of cells

Bone marrow-derived dendritic cells (BM-DC) were prepared as described elsewhere [21] and were used on day 7. Purity was routinely about 70%. BM-DC were incubated for 30 min in PBS with 1 µM GP33 peptide (DC-GP33) or with 0.1 µM adenovirus peptide at 37°C and washed, or left unpulsed.

Splenocytes of P14 mice were isolated by passing the spleen through a wire-mesh. Erythrocytes were lysed with 0.75% ammonium chloride, 0.21% Tris, pH 7.2 for two minutes at room temperature, followed by extensive washing. P14 Thy1.1^+^ T cells represented about 24% of total splenocytes.

### Protective DC vaccination for melanoma defense and analysis of proliferation state of effector T cells

A total of 1.5×10^6^ P14.1.1 splenocytes in 200 µl PBS were injected into the tail vein of Thy1.2^+^ C57BL/6 recipient mice (adoptive transfer). 3×10^5^ of either pulsed or unpulsed BM-DC were injected (i) intracutaneously (i.c.), (ii) intraperitoneally (i.p.) or (iii) intravenously (i.v.) into recipient mice. 1×10^6^ cells of each melanoma cell line were injected subcutaneously (s.c.), the parental cells (B16.F10) into the left flank and the GP33 expressing cells (B16.F10_GP33_) into the right flank of the same mice.

In the first protocol P14 transfer was performed on day 0, followed by BM-DC injection twice, on days 1 and 3, and both tumor cell lines (B16.F10 and B16.F10_GP33_) were inoculated on day 7. Protocol 2 to 4 did not include P14 transfer. In protocol 2 DC injection was performed twice, on days 0 and 2 with both tumor cell lines inoculated on day 4. Protocol 3 included only inoculation of GP33 expressing tumor on day 4 after repeated DC injection (day 0 and day 2). In protocol 4 a single DC injection was performed and only B16.F10_GP33_ was injected s.c. In protocol 5 a P14 transfer was performed on day 0 followed by a single DC injection on day 1. The proliferation state of P14.1.1 T cells was measured in the spleen and the expression of homing receptors on those cells isolated from the blood was measured on day 8. For all protocols see time axis in the corresponding figures.

### Antibodies and flow cytometry

All antibodies were used FITC, PE, Cy3, PE-Cy5, PE-Cy7, APC, eFluor660 or biotin conjugated. The biotinylated mAb were revealed with Streptavidin-PE-Cy5. mAb were used at 0.1–1 µg/1×10^6^ cells in 100 µl PBS/2% FCS/0.02% NaN_3_ (FACS buffer) or HBSS/0.3% BSA incubated on ice for 20–30 min. Antibodies used were: anti-CD16/CD32 (Fcγ III/II Receptor) (2.4G2), anti-CD90.1 (Thy1.1) (HIS51), anti-CD8α(Ly-2) (53–6.7), anti-CD3ε (145-2C11), anti-Vα2 T cell receptor (B20.1), anti-α4β7 (DATK32), anti-IA^b^ (AF6-120.1) and anti-CD11c (HL3) all from BD Biosciences (Heidelberg, Germany), anti-CCR9 (B cell hybridoma supernatant, kindly provided by Prof. R. Förster), anti-rat IgG (secondary for CCR9) (from Jackson ImmunoResearch Europe Ltd.), anti-CCR10 (248918), E-selectin/human IgG-Fc-Chimera (both from R&D) and polyclonal rabbit anti-human IgG (secondary for E-lig staining) (DakoCytomation, Hamburg, Germany).

All stainings were performed following a standard protocol except of E-lig staining. This staining was performed as described [16]. Control staining for E-lig and CCR9 was done with secondary Ab only, while corresponding isotype control mAb or fluorescence minus one (FMOs) were used as a control for all other stainings.

Data were acquired and analysed on a FACScan using CellQuestPro software (BD Biosciences) or on a FACS Canto II (Becton Dickinson) and analysed using FlowJo.

### Statistics

Statistical comparisons were performed by one-way ANOVA or a student *t* test using GraphPad Prism software. Unless stated otherwise, data are presented as means ± SEM of three to five replicates per experiment and each experiment is performed three times. Differences between groups were statistically significant at p<0.05.

## Results

### Melanoma protection is independent of DC vaccination route after adoptive transfer of TCR transgenic T cells

Previously our group was able to show that DC injected via different routes activate CD8^+^ T cells at different sites and imprint homing receptors corresponding to the site of priming [16] We were now interested if these differently primed T cells are able to migrate to distinct target tissues *in vivo* and if they are able to prevent subcutaneously (s.c.) growing melanoma with different potencies.

**Figure 1:**
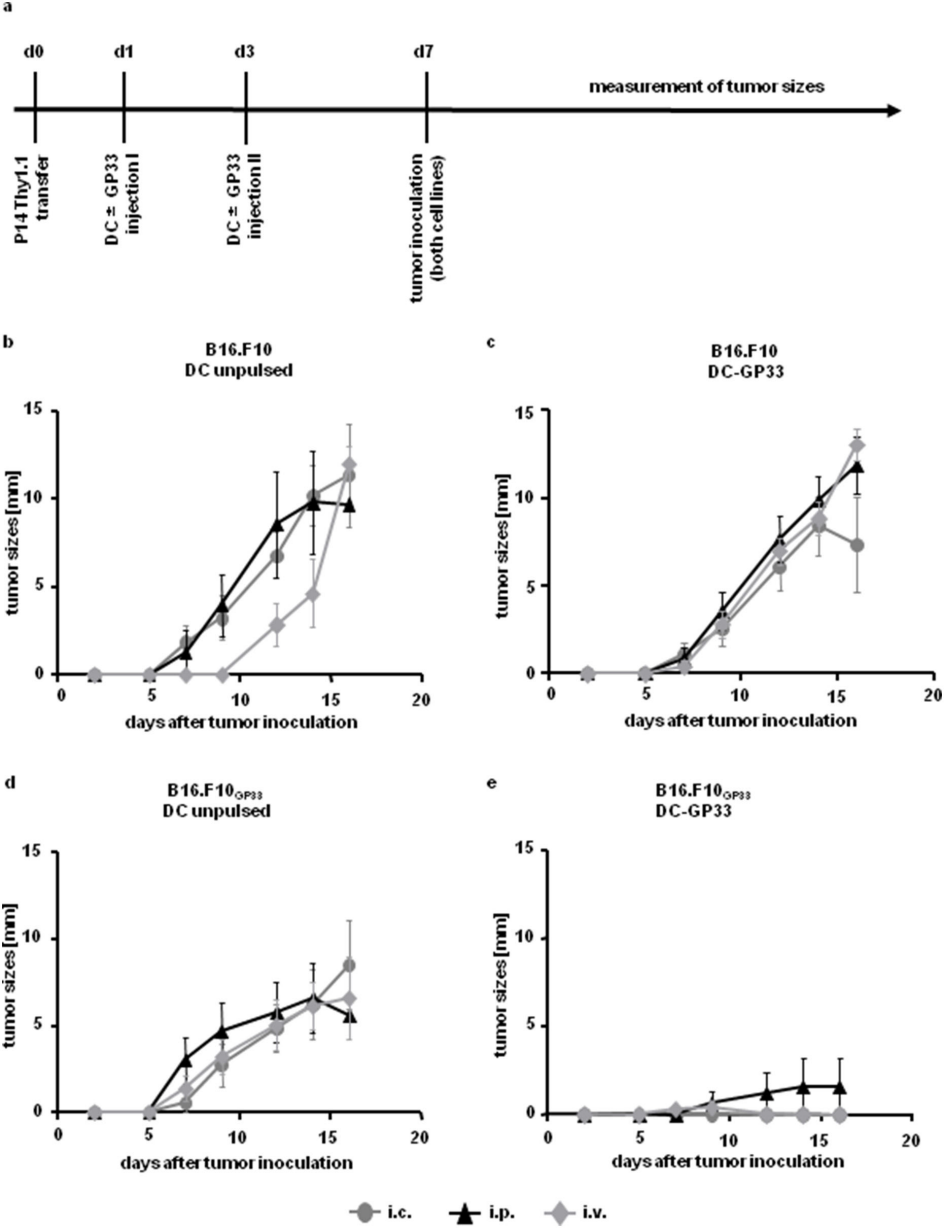
Tumor growth after T cell transfer and repeated DC vaccination via different injection routes. Time axis of experimental set up (*a*). 1.5×10^6^ Thy1.1^+^ P14 splenocytes were adoptively transferred i.v. into C57BL/6 (Thy1.2^+^) mice. One and three days later 3×10^6^ BM-DC, either pulsed (*c & e*) or left unpulsed (*b & d*) were injected (i) intracutaneously (i.c.) (*grey cycles*), (ii) intraperitoneally (i.p.) (*black triangles*) or (iii) intravenously (i.v.) (*grey diamonds*). On day seven after P14 transfer 1×10^6^ cells of the melanoma cell lines B16.F10 (*b & c*) and B16.F10_GP33_ (*d & e*) were inoculated subcutaneously (s.c.) into the left and the right flank, respectively. Every second day tumor sizes were measured. Tumor diameters are depicted. Three independent experiments were performed with 4 mice in each group. Error bars: SEM.

We started this approach by adoptively transferring splenocytes from T cell receptor transgenic P14 mice (Thy1.1^+^) into C57BL/6 recipient mice (Thy1.2^+^). Peptide pulsed or unpulsed BM-DC were injected either intravenously (i.v.), intraperitoneally (i.p.) or intracutaneously (i.c.) twice, one and three days after T cell transfer. Four days after the last BM-DC injection, a parental melanoma cell line B16.F10 was inoculated s.c. in the left flank and the same tumor, but expressing the peptide GP33 (B16.F10_GP33_) was injected s.c. in the right flank of the same mouse (Fig. 1a). Tumor sizes were measured every second day. The parental tumors always grew quickly irrespectively of the immunization route and peptide loading of BM-DC (Fig. 1b & 1c). B16.F10_GP33_ grew in mice injected with unpulsed BM-DC, irrespectively of the injection route (Fig. 1d). In contrast DC-GP33 were able to prevent tumor growth after i.c. and i.v. injection (Fig. 1e), reflecting a significant difference to non- pulsed BM-DC after i.c. and i.v. vaccination from day 12 after tumor inoculation. I.p. injection of DC-GP33 reduced tumor growth compared to unpulsed BM-DC injection (Fig. 1e).

Adoptively transferring less P14 T cells (as little as 5×10^2^ splenocytes) followed by repeated DC vaccination and tumor inoculation resulted in GP33 rejection of B16.F10_GP33_ independent of the injection route of DC-GP33 (data not shown).

### Efficiency of tumor defense depends on the DC vaccination route

**Figure 2:**
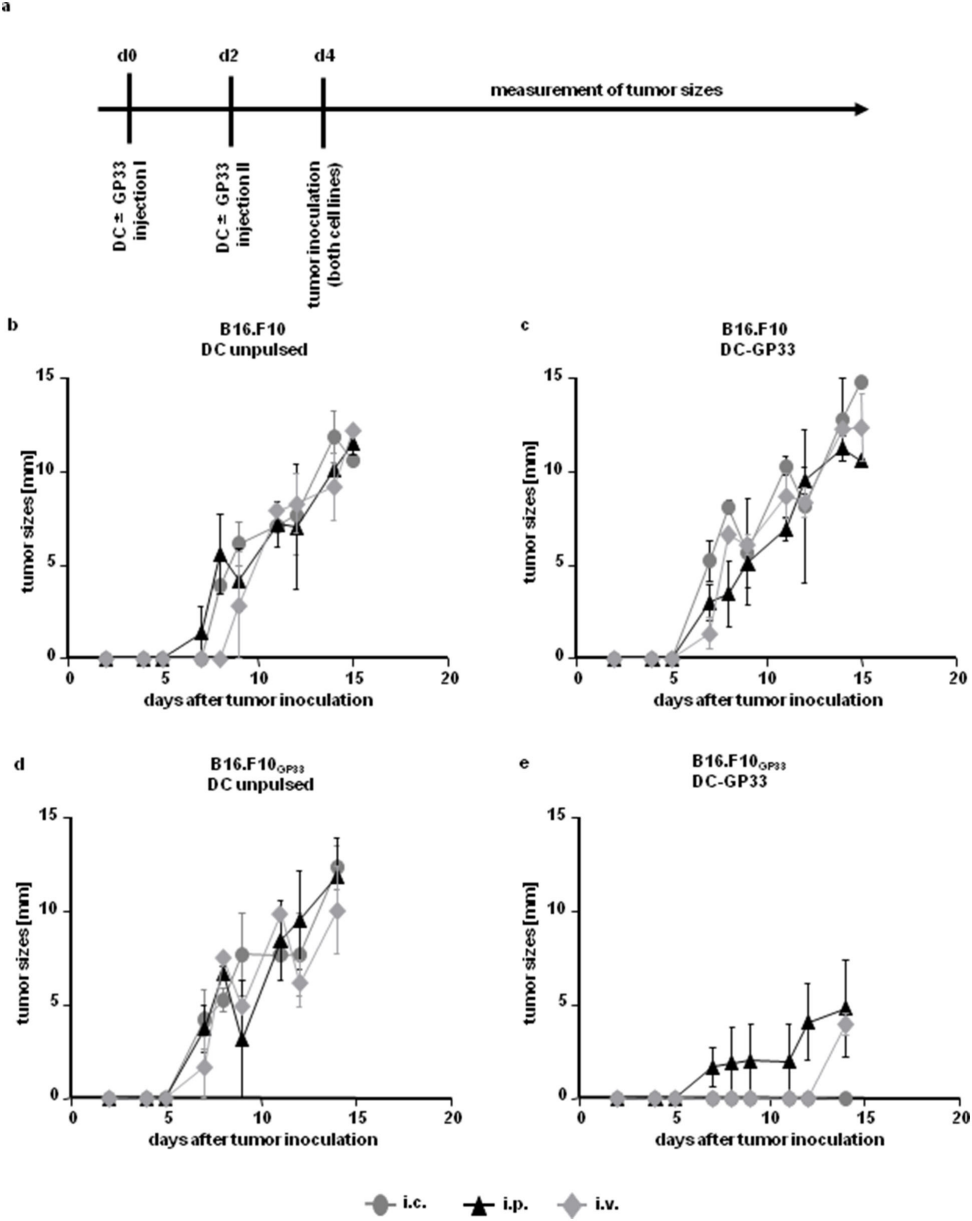
Tumor growth after repeated DC vaccination without T cell transfer. Time axis of experimental set up (*a*). 3×10^6^ BM-DC were injected into C57BL/6 recipient mice twice, either peptide pulsed (*c & e*) or unpulsed (*b & d*) via the (i) i.c. (*grey cycles*), (ii) i.p. (*black triangles*) or (iii) i.v. (*grey diamonds*) route on day 0 and day 2. Two days later 1×10^6^ cells of both tumor cell lines were injected s.c. into the left and the right flank, respectively. Tumor sizes were measured every second day. Diameters of tumors are shown. N = 6 per group, from two independent experiments. Error bars: SEM.

Since GP33 is a potent antigen that induces robust T cell responses in C57BL/6 mice [30]–[32] we decided to go on without transfer of P14 splenocytes but continued to inject BM-DC twice via the three different injection routes and tumor inoculation at day 4 (Fig. 2a). Again all tumors grew if BM-DC were left unpulsed and injected via any route (Fig. 2b & 2d). DC-GP33 injection did not lead to rejection of the parental tumor (Fig. 2c). In contrast, i.p. injection of DC-GP33 dampened, and i.v. injection delayed the onset of GP33 expressing tumor growth compared to controls. Intracutaneous application of DC-GP33 resulted in a complete tumor rejection, presenting a significant difference to unpulsed BM-DC injection via any route on day 14 after tumor inoculation (Fig. 2e).

### Growth of GP33 expressing tumor is diminished after repeated DC vaccination in mice with lower tumor burden

**Figure 3:**
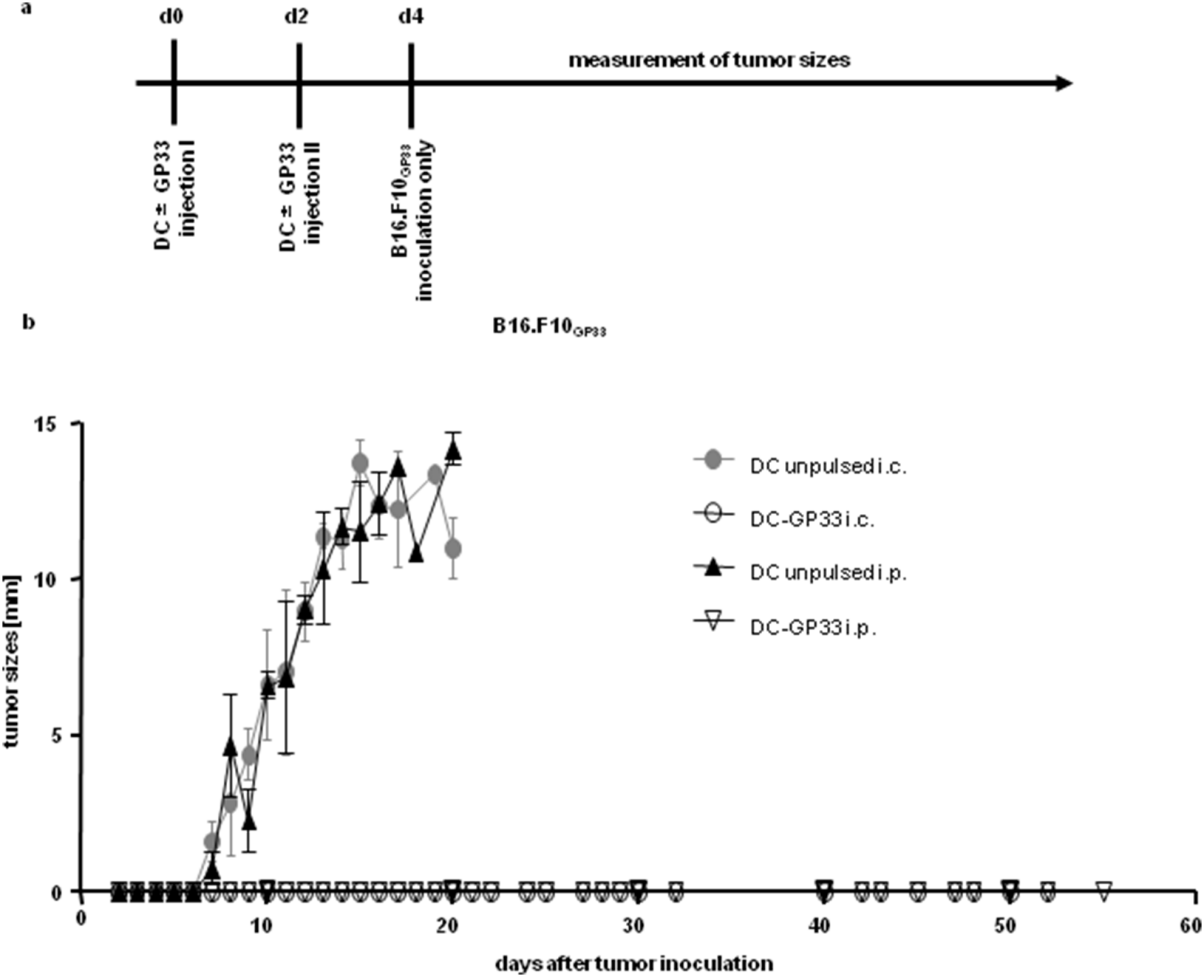
Growth of GP33-expressing tumor only after repeated DC vaccination. Time axis of experimental set up (*a*). BM-DC (3×10^6^) were injected twice into C57BL/6 recipient mice, either peptide-pulsed (*open symbols*) or unpulsed (*filled symbols*) via the (i) i.c. (*cycles*) or (ii) i.p. (*triangles*) route on day 0 and day 2. Two days later 1×10^6^ cells of the GP-33 expressing tumor cell line were injected s.c. into the left flank (*b*). Tumor sizes were measured every second day. Diameters of tumors are shown. Four independent experiments were performed with n = 5 mice per group in three experiments and n = 3 in one experiment. Error bars: SEM.

According to animal welfare regulations mice have to be sacrificed as soon as the tumor reaches a diameter of 15 mm. In order to examine the ability of T cells to reject tumors after DC vaccination for a longer period of time we performed similar experiments, injecting DC-GP33 or unpulsed BM-DC twice, either i.p. or i.c. but only inoculated the GP33 expressing tumor s.c (Fig. 3a). During long term observations the tumor grew normally in control mice (which have received unpulsed BM-DC either i.p. or i.c.) but interestingly no tumor was palpable if DC-GP33 were injected either i.c. or i.p. (Fig. 3b). Therefore a rather low tumor burden (compared to earlier experiments, where both tumor cell lines were injected) led to long-term rejection of tumor growth independent of the DC vaccination route after repeated DC inoculation.

### After a single DC vaccination only intracutaneous DC inoculation induces long-term prevention of tumor growth

**Figure 4:**
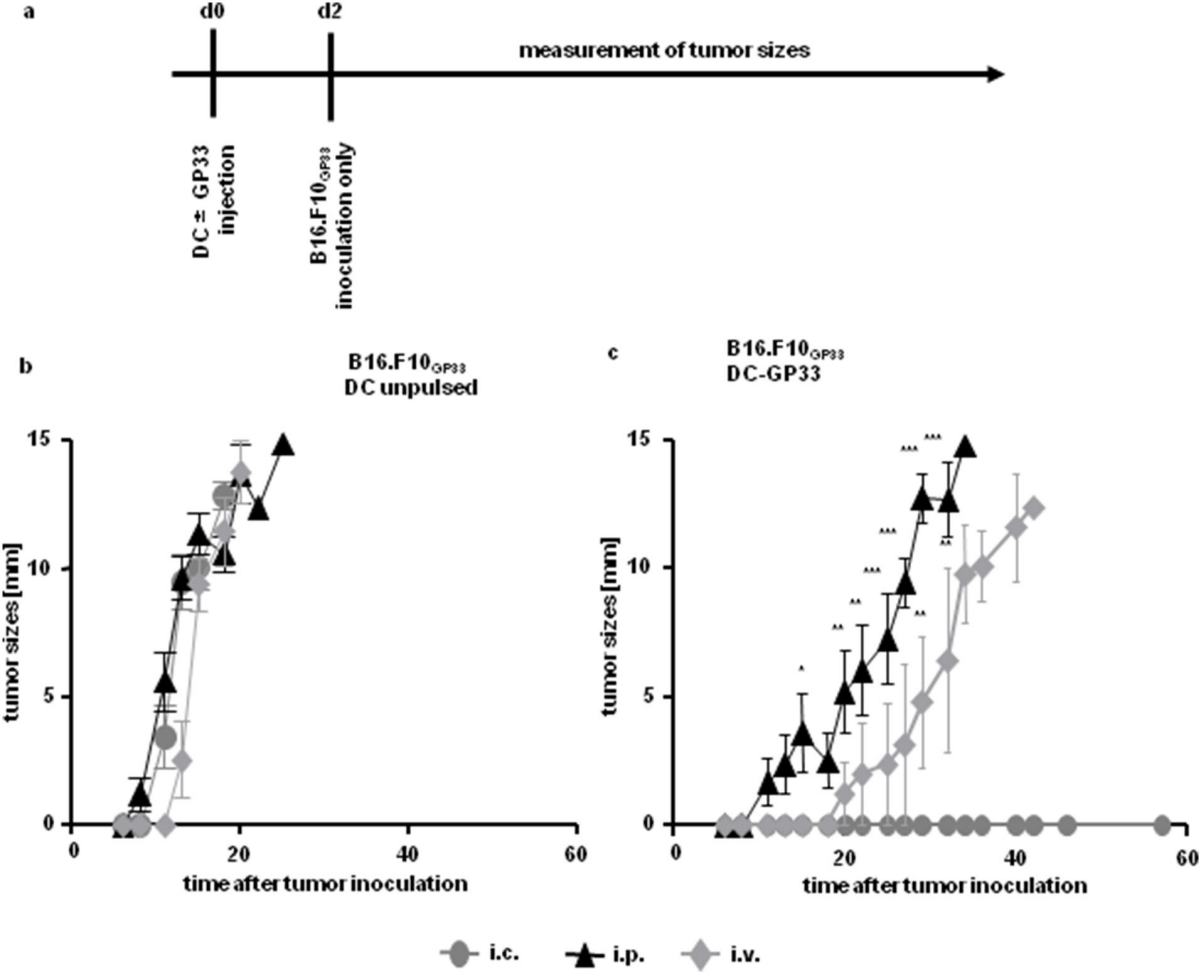
Growth of GP33-expressing tumor after a single injection of BM-DC. Time axis of experimental setup (*a*). 3×10^6^ BM-DC either loaded with peptide (*c*) or left unpulsed (*b*) were injected once (i) i.c. (*cycles*), (ii) i.p. (*triangles*) or (iii) i.v. (*diamonds*). Two days later 1×10^6^ cells of the tumor cell line B16.F10_GP33_ was inoculated s.c. and tumor sizes were measured every second day for up to 56 days. Diameters of tumors are depicted. Figure shown for i.c. and i.p. injection route of BM-DC included 13 animals in total, with n = 5 animals per group in two experiments and n = 3 animals in one experiment. Figure including the i.v. vaccination route include five animals in total from one experiment. Error bars: SEM.

We continued with experiments injecting BM-DC only once, either peptide pulsed or unpulsed, again via all three injection routes. Two days later B16.F10_GP33_ cells were inoculated s.c. into the flank (Fig. 4a). Unpulsed BM-DC did not mount an immune response against s.c. growing melanoma and the tumor grew irrespective of the injection route of BM-DC (Fig. 4b). DC-GP33 injected i.p. or i.v. were not able to induce a sufficient immune response against tumor located subcutaneously either, resulting in a delayed onset of melanoma growth compared to control mice (Fig. 4c). The i.p. and i.v. vaccination routes led to palpable tumors starting at day 11 and day 22 respectively. In contrast the i.c. vaccination route completely diminished tumor growth over a long period of time (total time of observation: 57 days after tumor inoculation) in all mice. From day 15 after tumor inoculation onwards the i.p. vaccination route results in tumor growth with a statistical difference compared to the i.c. vaccination route. The i.v. immunization route led to tumor outgrowth with a statistical difference from day 29 onwards, compared to the i.c. vaccination route of DC-GP33.

### Differential homing receptor imprinting on T cells and differential proliferation state depending on the DC vaccination route

We hypothesized that activated cytotoxic effector T cells migrate into the skin and hence are able to fight tumor located s.c. only after i.c. vaccination. To address this we analyzed the expression patterns of homing receptors on effector T cells *ex vivo*.

**Figure 5:**
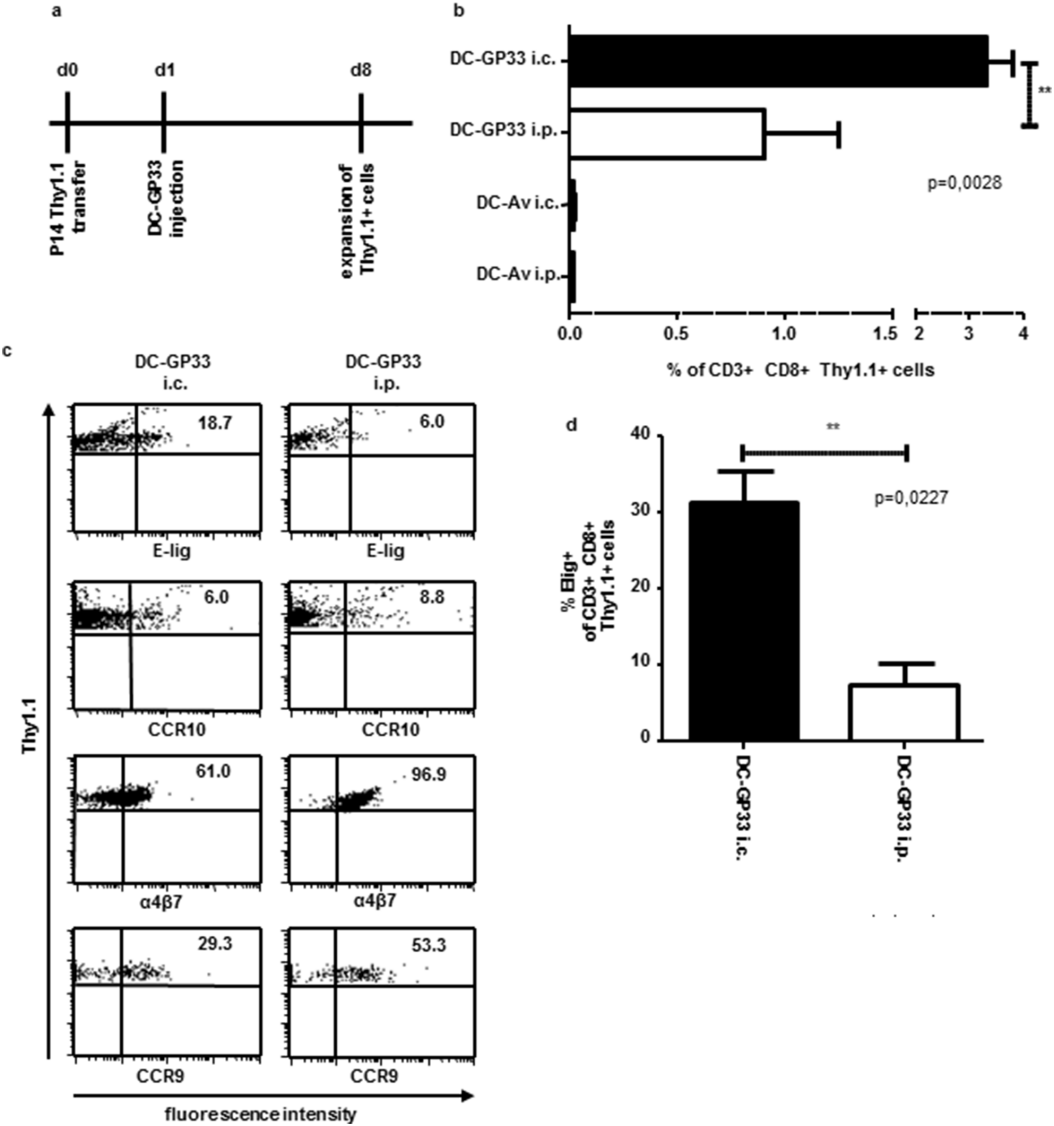
Proliferation and homing receptor expression on tumor specific T cells after DC vaccination via different injection routes. Time axis for experimental set up (*a*). 1.5×10^6^ Thy1.1^+^ P14 splenocytes were adoptively transferred i.v. into C57BL/6 (Thy1.2^+^) mice. One day later 3×10^6^ DC-GP33 or DC-Av were injected (i) i.c. or (ii) i.p. On day 8 after P14 transfer mice were sacrificed and cells from the spleen (*b & d*) and blood (*c*) were analyzed. Mean population size of CD3^+^CD8^+^Thy1.1^+^ cells from spleen are depicted (*b*) significant difference was calculated using an unpaired *t*-Test after i.c. or i.p. DC-GP33 vaccination route. N = 6 mice, error bars: SEM. One representative analysis of homing receptor expression on Thy1.1^+^ T cells in the blood after DC-GP33 vaccination via the i.c. or the i.p. route is shown. Homing receptors are specified below each panel (*c*). Numbers in dot blots indicate % of positive cells. Skin homing receptor E-lig expression was measured via FACS analysis in the spleen after DC-GP33 vaccination via the i.c. or the i.p. route (*d*). Mean E-lig expression level of CD3^+^CD8^+^Thy1.1^+^ cells are depicted from n = 3 mice. Three mice were included in every group per experiment, two (*b & d*) and three (*c*) independent experiments were performed.

As the frequency of endogenous effector T cells was too small to be analyzed by MHC tetramer staining and homing receptor analysis (unpublished data) we injected P14.1.1 splenocytes one day prior to a single peptide pulsed DC vaccination, which allowed analysis of expansion and homing receptor expression *ex vivo* on the TCR transgenic Thy1.1^+^ T cells. We loaded DC either with the GP33 peptide or, as control, with an irrelevant adenovirus peptide (Av). With the congenic marker, Thy1.1, transferred T cells could later be distinguished from endogenous T cells. Thy1.1^+^ T cells from the spleen, blood (Fig. 5) and skin draining inguinal lymph nodes (data not shown) were analyzed 7 days after DC inoculation. The vaccination route of DC-GP33 had an impact on the size of the effector T cell population as the percentage (Fig. 5b) and the absolute numbers (data not shown) of Thy1.1^+^ cells in the spleen were significantly higher after i.c. injection compared to the i.p. route. DC loaded with the adenovirus peptide did not lead to a proliferation of Thy1.1^+^ P14 T cells (Fig. 5b). Induction of the skin homing receptor E-selectin ligand was increased on Thy1.1^+^ T cells in the blood and spleen, after i.c. injection of DC-GP33 compared to the i.p. vaccination route (Fig. 5c, d). In contrast CCR10 expression on T cells was minimal but comparable in the blood after the i.c. injection route of DC-GP33 compared to the i.p. route (Fig. 5c). The small intestine homing receptors α4β7-integrin and CCR9 were increased after i.p. injection of BM-DC compared to i.c. injection in the blood, although both receptors also could be detected upon i.c. vaccination (Fig. 5c). Taken together these results indicate that the injection route of DC-GP33 and the efficiency impacts the induction of tissue-specific homing receptors on effector T cells.

## Discussion

One reason for the limitations of cancer immunotherapy by DC-based vaccination is the immune evasion strategies of tumors. Our observation that the DC injection route impacts on the homing receptor imprinting on T cells [16] indicates that another reason for the limited success of DC based immunotherapy against melanoma might be the choice of injection route. Thus the efficiency of the anti-tumor response may be critically influenced by the targeted migration of effector T cells to the tumor site as guided by the DC vaccination route. In line with this, Fong and colleagues reported that DC administered via different routes to patients with metastatic prostate carcinoma prime T cell immunity irrespectively of the injection route. However, only intradermal and intralymphatic DC vaccination resulted in IFN-γ production whereas i.v. injection caused an IL-4 response and the induction of antigen specific antibodies [8]. In line with our previous observations [16] and with this study, Grover et al. injected DC pulsed with melanoma antigens intralymphatically in the dorsal side of the foot of patients with metastatic melanoma [33]. Using this route a regression of cutaneously localized disease was observed. Most importantly, T cells expressing CLA and CCR6 were induced via this route. These findings indicate that optimal T cell trafficking to tumor sites depends on the vaccination route in DC immunotherapy.

It has to be considered that only CCR7^+^ mature antigen-pulsed DC efficiently migrate to the draining LN and induce T cell proliferation [34] and that the route of DC injection determines the distribution of effector and memory T cells [15], [35]–[37]. Since *in vitro* generated DC do not imprint tissue specific homing receptor profiles on T cells *in vitro*, it must be assumed that the DC acquire this potential in the tissue that they are injected into, or alternatively, in the lymph nodes. Calzascia and colleagues showed that the tissue origin and site of antigen encounter of DC is a crucial parameter in homing receptor imprinting [25]. Furthermore, we were able to show that peripheral tissue cells influence the capacity of DC to induce different homing receptors on T cells [22]. Moreover, LN stromal cells are also involved in the induction of specific homing receptors on T cells [23], [24]. Thus, both the peripheral and lymph node tissue microenvironments have an effect on the capacity of DC to induce specific homing receptors on T cells. In the case of *in vitro* generated DC, therefore, the injection route is important with respect to the site of T cell priming and homing receptor imprinting.

Here we describe that adoptive transfer of GP33 specific P14.1.1 T cells and repeated DC injection prior to tumor inoculation resulted in an efficient protective anti-tumor response that was independent of the vaccination route. This was possibly due to a non-physiologically high frequency of tumor-specific effector T cells, both transgenic and endogenous. Surprisingly as little as 5×10^2^ transferred P14.1.1 splenocytes still exhibited this effect supporting a high frequency of endogenous GP33 specific CD8^+^ T cell precursors [38] and the strong immunogenicity GP33 in C57BL/6 mice as reported by Butz et al and Murali-Krishna et al [30], [31]. It was further demonstrated that LCMV-immune effector cells from C57BL/6 mice are able to lyse B16.F10_GP33_ efficiently [29].

Systemic T cell responses following local priming have been observed by others. In fact it has been shown that local virus infections can result in a systemic immune response. Oral infection of mice with the EDIM strain of rotavirus results in an acute intestinal infection. Despite the gut-restricted infection, activated CD8^+^ T cells were found to migrate to non- lymphoid sites beyond intestinal tissue [39]. Furthermore, the widespread migration of effector T cells was not dependent on the inflammation associated with the infection. This was shown by using a line of transgenic mice expressing OVA specifically in small intestinal mature enterocytes. After adoptive transfer of OT-I/RAG^−/−^ CD8^+^ T cells they found those cells predominantly in Peyer’s patches, the Lamina propria and as intraepithelial lymphocytes. Besides these locations, the OT-I cells were also found in all other tissues examined [39].

Even without P14 transfer, tumor growth was reduced but measurable after repeated i.p. injection of DC-GP33 compared to controls. This is in line with studies by Zitvogel who demonstrated suppression of tumor growth independent of the DC vaccination route upon repeated DC vaccinations [40]. Only if we injected DC-GP33 twice via the i.c. route the GP33-expressing tumor growth was diminished completely, after inoculation of both tumor cell lines. In this experimental set-up i.v. injection led to a delayed onset of melanoma growth. This could be due to an escape mechanism of the tumor, e.g. by down-regulation of the tumor antigen (in this case GP33) and thus lack of recognition by GP33-specific effector T cells, although this was only reported in very rare cases [29]. The difference seen after i.p. and i.c. injection, regarding tumor growth, suggests that the efficiency of the protective T cell response with respect to the different vaccination routes was due to differential induction of homing receptors on effector T cells as well as differential proliferation state of effector T cells due to different vaccination routes.

When we monitored melanoma growth for a longer period of time, no tumor was palpable after repeated i.p. injection of DC-GP33 when only B16.F10_GP33_ was inoculated. This was in contrast to our observation that i.p. injection did not efficiently prevent tumor growth when both the parental and the GP33 expressing B16.F10 cells were inoculated. This may be an effect of overall tumor burden possibly due to an immunosuppressive function of the tumors, e.g. due to the production of immunosuppressive cytokines like TGFβ [41] and IL-10 [42] or the expression of Fas ligand on the tumor cell surface [43]. Inoculation of B16.F10_GP33_ only may thus reduce the amount of immunosuppressive molecules. Therefore the vaccination route became irrelevant and hence the i.p. immunization route was also able to sufficiently activate CD8^+^ T cells and prevent tumor growth.

When mice were vaccinated only once with DC and, therefore, a boosting effect for CD8^+^ T cells was missing, now the role of the immunization routes became apparent. DC-GP33 suppressed tumor growth completely after i.c. injection whereas neither i.p. nor i.v. immunization could prevent tumor growth. Compared to control mice tumor growth was delayed in mice which received DC-GP33 i.p. or i.v. This indicates that peptide-loaded DC activated CD8^+^ T cells, irrespective of the immunization route (confirming data from Fong et al [8]), but that only the i.c. route led to tumor rejection, either by licensing effector T cells to migrate into the skin efficiently and/or due to an enhanced expansion effector T cells. These results are in accordance with the report from Masopust et al. showing a systemic immune response to a local infection [39]. Hence the migration of effector T cells to other sites than the local lymph node and/or tissue of origin of the DC may explain our findings using repeated DC vaccination. It remains to be determined if this correlates with the extent of expansion of the T cell pool and/or different homing receptor profiles induced following a single or multiple DC vaccinations. Alternatively, repeated i.p. or i.v. DC vaccinations may result in the saturation of the draining lymph nodes and spleen and localization of the antigen-loaded DC also in peripheral skin-draining lymph nodes. This saturation effect has been discussed by Mullins [15]. In this study DC were injected only once subcutaneously and detected not only in the axillary lymph nodes but also in the spleen. This was interpreted as a potential saturation of the draining lymph node by small numbers of injected DC. Future experiments must address these alternative possibilities in systems where DC and antigen-specific T cells can be traced. Our results further strengthen our hypothesis that the application route of DC-GP33 is important for the potential to prevent s.c. melanoma growth.

Adoptive transfer of congenic P14.1.1 splenocytes followed by a single DC-GP33 vaccination (either i.c. or i.p.) resulted in unequal frequencies of effector CD8^+^ T cells in the spleen. These results show that the expansion of effector T cells was at least in part also responsible for the differential outcome of tumor growth after DC vaccination via different routes.

Additional to the effects in proliferation, we observed a differential induction of one of the skin homing receptors on effector T cells. Compared to the i.p. vaccination route the i.c. injection of DC-GP33 led to an enhanced expression of E-lig on Thy1.1^+^ T cells in the (Fig. 5c) blood and spleen (Fig. 5d) as well as in skin draining inguinal LN (data not shown). The induction of the skin homing receptor CCR10 was not as distinct. It was also elevated on effector T cells in the skin draining LN after i.c. vaccination but comparably expressed in the blood after i.c. or i.p. vaccination. The expression of the small intestine homing receptors α4β7 and CCR9 were increased on T cells in the blood after i.p. immunization confirming other studies [15], [35]. These data indicate that the i.c. vaccination route is more efficient in activating and licensing tumor specific CD8^+^ T cells to migrate into the skin and hence prevent subcutaneous tumor growth.

In conclusion our experiments indicate that the vaccination route of DC is crucial for the *in vivo* proliferation and induction of distinct homing receptor profiles on effector T cells and therefore for their ability to migrate into peripheral target tissues and tumors. This may be especially important in the clinical situation of an established tumor that reduces the efficiency of immune responses by evasion strategies. This suboptimal situation may be reflected in our experiments when DC were injected only once. Therefore the imprinting of tissue specific homing receptors on effector T cells might be pivotal in the outcome of the tumor immunotherapy and strategies that manage to optimize T cell homing to tumors should improve efficacy of DC-based cancer immunotherapies [44].
